# Novel Regulatory Mechanisms for Generation of the Soluble Leptin Receptor: Implications for Leptin Action

**DOI:** 10.1371/journal.pone.0034787

**Published:** 2012-04-24

**Authors:** Michael Schaab, Henriette Kausch, Juergen Klammt, Marcin Nowicki, Ulf Anderegg, Rolf Gebhardt, Stefan Rose-John, Juergen Scheller, Joachim Thiery, Juergen Kratzsch

**Affiliations:** 1 Institute of Laboratory Medicine, Clinical Chemistry and Molecular Diagnostics, University of Leipzig, Leipzig, Germany; 2 Hospital for Children and Adolescents, University of Leipzig, Leipzig, Germany; 3 Institute of Anatomy, Faculty of Medicine, University of Leipzig, Leipzig, Germany; 4 Department of Dermatology, Venerology and Allergology, Faculty of Medicine, University of Leipzig, Leipzig, Germany; 5 Institute of Biochemistry, Faculty of Medicine, University of Leipzig, Leipzig, Germany; 6 Institute of Biochemistry, Christian-Albrechts-University Kiel, Kiel, Germany; University of Tor Vergata, Italy

## Abstract

**Background:**

The adipokine leptin realizes signal transduction via four different membrane-anchored leptin receptor (Ob-R) isoforms in humans. However, the amount of functionally active Ob-R is affected by constitutive shedding of the extracellular domain via a so far unknown mechanism. The product of the cleavage process the so-called soluble leptin receptor (sOb-R) is the main binding protein for leptin in human blood and modulates its bioavailability. sOb-R levels are differentially regulated in metabolic disorders like type 1 diabetes mellitus or obesity and can, therefore, enhance or reduce leptin sensitivity.

**Methodology/Principal Findings:**

To describe mechanisms of Ob-R cleavage and to investigate the functional significance of differential sOb-R levels we established a model of HEK293 cells transiently transfected with different human Ob-R isoforms. Using siRNA knockdown experiments we identified ADAM10 (A Disintegrin And Metalloproteinase 10) as a major protease for constitutive and activated Ob-R cleavage. Additionally, the induction of lipotoxicity and apoptosis led to enhanced shedding shown by increased levels of the soluble leptin receptor (sOb-R) in cell supernatants. Conversely, high leptin concentrations and ER stress reduced sOb-R levels. Decreased amounts of sOb-R due to ER stress were accompanied by impaired leptin signaling and reduced leptin binding.

**Conclusions:**

Lipotoxicity and apoptosis increased Ob-R cleavage via ADAM10-dependent mechanisms. In contrast high leptin levels and ER stress led to reduced sOb-R levels. While increased sOb-R concentrations seem to directly block leptin action, reduced amounts of sOb-R may reflect decreased membrane expression of Ob-R. These findings could explain changes of leptin sensitivity which are associated with variations of serum sOb-R levels in metabolic diseases.

## Introduction

Leptin (Ob), a 16 kDa protein primarily secreted by adipocytes, regulates energy homeostasis via so called central mechanism in the brain. Besides its central effects in the brain, a large number of peripheral actions have been discovered in the last years. Thus, leptin affects the immune system by influencing CD4+ T-cell polarization, B-cell homeostasis as well as renal macrophage infiltration [1]–[3] and modulates the severity of sepsis [4]. Furthermore, leptin plays a role in the regulation of liver and skeletal muscle lipid oxidation and glucose metabolism [5]–[9]. Leptin actions are mediated through the leptin receptor (Ob-R) that belongs to the class I cytokine receptor family [10]. So far in humans four different membrane-anchored Ob-R isoforms have been discovered which have identical extracellular, ligand-binding and transmembrane domains but differ in the length of the intracellular domain. Only the long form (Ob-Rfl) has the so called Box 3 motif with Tyr_1141_ and is therefore considered to possess full signaling capacity via the JAK-STAT (Janus kinase-signal transducer and activator of transcription) pathway [11]. However, latest findings indicate that short isoforms may be involved in leptin signaling as well [12]. In addition, the presence of a soluble receptor form (sOb-R) [13], [14] was demonstrated in peripheral blood that represents the main leptin binding activity [15] and reflects the density of membrane Ob-R [16]. Previous findings suggest a predominant inhibitory effect of sOb-R on leptin bioactivity in cell and animal models [17], [18]. Thus, high sOb-R concentrations neutralized leptin-mediated STAT3 signaling and anorexic responses in rats [19] and protected mice against experimentally-induced sepsis [4]. Interestingly, transgenic mice with an overexpression of sOb-R showed decreased body weight and increased energy expenditure which would point to a positive correlation between serum sOb-R concentrations and leptin sensitivity [20]. Clinical studies demonstrated both increased and decreased levels of sOb-R according to the metabolic situation of the patient. A lack of substrates like glucose in the cellular energy stores as shown in newly manifested type 1 diabetes mellitus (T1DM) or anorexia nervosa is associated with an up to a 100-fold molar excess of sOb-R over leptin in blood [21], [22]. In newly manifested T1DM, lipotoxic effects by elevated levels of free fatty acid (FFA) may finally cause tissue specific cell death through apoptosis [23]–[26] and could be of general importance for the shedding process. In contrast, excess of substrates like glucose in the energy stores, as in obesity, was associated with diminished serum levels of sOb-R. In addition, a strong inverse correlation between plasma sOb-R levels and the risk of developing type 2 diabetes mellitus in human patients points to an important role of sOb-R as modulator of leptin action [16], [27]–[29]. Although having increased leptin concentrations many of obese patients are resistant to leptin action. Ob-R downregulation besides other factors like suppressor of cytokine signaling 3 (SOCS3) and protein-tyrosine phosphatase 1B (PTP1B), could be involved in the pathogenesis of leptin resistance [30]–[33]. However, the molecular mechanisms for receptor downregulation remain completely unclear. Endoplasmic reticulum (ER) stress is a recently identified key mechanism for the development of obesity and leptin resistance [34]–[36] and therefore could be a trigger for Ob-R downregulation and suppressed sOb-R generation. The aim of this work was to investigate regulatory mechanisms of increased and suppressed generation of sOb-R in a cell model and to elucidate proteases which process the extracellular Ob-R domain. By employing specific inhibitors and siRNA technique we demonstrate the involvement of ADAM10 and even though to a minor degree of ADAM17 in constitutive and activated Ob-R shedding. Moreover we reveal that apoptosis and lipotoxicity induce processing of the extracellular Ob-R domain. In addition, we show that ER stress and high but still physiological leptin concentrations decrease sOb-R concentration in the cell supernatant. These data present new insights into regulatory mechanisms of sOb-R generation and add new knowledge to the concept of leptin resistance.

## Materials and Methods

Phorbol myristate acetate (PMA), N-[(2R)-2-(hydroxamidocarbonylmethyl)-4-methylpentano-yl]-L-tryptophan methylamide (GM6001), staurosporine and Z-VAD(OMe)-FMK were purchased from Calbiochem (Merck, Darmstadt, Germany). Fetal bovine serum and PBS were purchased from Biochrom (Biochrom, Berlin, Germany). DMEM/F12, Opti-MEM, penicillin and streptomycin, glutamine as well as Lipofectamine 2000 and Lipofectamine RNAiMAX were purchased from Invitrogen (Invitrogen by Life Technologies, Darmstadt, Germany). Dimethyl sulfoxide, sodium palmitate, –oleate, bovine serum albumin and fatty acid free bovine serum albumin (BSA) were purchased from Sigma Aldrich (Sigma Aldrich, Munich, Germany). Metalloprotease inhibitors GI254023X (GI) and GW280264X (GW) were kindly provided by Dr. JD Becherer (GlaxoSmithKline, Research Triangle Park, North Carolina 27709, USA). CCK8-Assay for determination of cell viability was purchased from Dojindo Laboratories (Dojindo Laboratories, Munich, Germany).

### Plasmid construction

cDNA plasmids for the human short Ob-R isoforms were generated by subcloning of the unique parts of the respective isoforms into pcDNA 3.1(+)/ObRfl (kindly provided by Prof. Ross University of Sheffield, UK). Furthermore we subcloned the cDNA for Ob-Rfl and Ob-R219.3 into pcDNA3.1 Zeo-myc to obtain Ob-R constructs myc-tagged at the N-terminus. Sequence integrity of the constructs was verified by restriction endonuclease digestion and sequencing. The myc-tagged constructs were used for all experiments except the investigations on the substrates for Ob-R shedding and the influence of leptin on constitutive shedding. Detailed information concerning the cloning of the different constructs is available on request.

### Cells culture and transient siRNA or plasmid transfection

Human embryonic kidney (HEK) 293 cells (American Type Culture Collection (ATCC) Number: CRL-1573) were cultured in DMEM/F12 supplemented with 10% fetal bovine serum, 100 U/ml penicillin, 100 µg/ml streptomycin and 2 mM glutamine. One day before transient transfection HEK293 cells were seeded into 6-well or 12-well plates in a density of 0.3×10^6^ cells or 0.15×10^6^ cells/well respectively. For siRNA experiments cells were seeded in a density of 0.2×10^6^ cells/6-well. On the day of transfection DMEM/F12 medium was replaced with Opti-MEM and HEK293 cells were transfected with Ob-R plasmids (1 µg) or siRNA (50 nM) using Lipofectamine 2000 or Lipofectamine RNAiMAX, according to the manufacturer's instructions. 24 hours after siRNA transfection the cells were transfected with the respective Ob-R constructs using Lipofectamine 2000. Stimulation experiments with the indicated substances were carried out in DMEM/F12 medium containing 100 U/ml penicillin, 100 µg/ml streptomycin and 2 mM glutamine, 0,1% BSA or 2% BSA (fatty acid free) for palmitate and oleate incubations. To exclude a possible influence of the solvent of the used substances controls were performed as indicated.

### RNA Isolation and Reverse Transcription

After aspirating the medium, total RNA from transiently transfected cells was isolated using Trizol Reagent (Invitrogen) according to the instructions of the manufacturer. Quality of the isolated RNA was confirmed by spectrophotometric measurement at 260 nm and 280 nm (ND 1000, Nano Drop Products, Wilmington, DE) and through gel electrophoresis. 2 µg of RNA were used for first strand DNA-synthesis using SuperScript II (Invitrogen) following the manufacturer's instruction. cDNA was diluted 1∶10 in Tris-EDTA-buffer and kept at −20 C until use.

### Primer, probes and siRNA

Primers (Eurofins MWG Operon, Ebersberg, Germany) were choosen to selectively amplify cDNA and to avoid amplification of genomic DNA traces. ADAM10 (Gen-Bank NM_001110): forward: 5′-CAAAGTCTGAGAAGTGTCGGG-3′, reverse: 5′-CTGCACATTGCCCATTAATG-3′, anneal-ing temperature 60°C, ADAM17 (GenBank NM_003183): forward: 5′-GAGGAAAGGAAA GCCCTGTACAGTAG-3′, reverse: 5′-CAGCTGGTCAATGAAATCCCAA-3′, annealing temperature 60°C, β-actin (GenBank NM_001101): forward: 5′-CCTGGCACCCAGCACAAT-3′, reverse: 5′-GCCGATCCACACGGAGTACTT-3′, annealing temperature 60°C. The applied fluorescent probes (Eurofins MWG Operon, Ebersberg, Germany) lie across an exon-exon boundary to further improve specificity of the product detection. Sequences were as follows: ADAM17: 5′-TTGTGACATGAATGGCAAATGTGAGAAACG-3′, β-actin: 5′-ATCA AGATCATTGCTCCTCCT GAGCGCA-3′. The following siRNAs specific for ADAM10 (Stealth Select RNAi HSS100167) or ADAM17 (Stealth Select RNAi HSS110436) were used in the present investigation and purchased from Invitrogen. The applied non-targeting siRNA (ON-TARGET*plus*, D-001810-01-20) was purchased from Dharmacon (Dharmacon, Lafayette, CO).

### Quantitative real-time PCR

For detection of ADAM10 mRNA expression we used SYBR Green I intercalating dye and for ADAM17 and β-actin mRNA expression TaqMan quantitative real-time PCR. 2.5 µl of the respective cDNA were amplified with the AmpliTaq Gold GeneAmp 5000 10 x PCR Buffer II & MgCl_2_ Solution Kit or the Power SYBR Green Master Mix (Applied Biosystems by Life Technologies, Darmstadt, Germany). The following experimental protocol was applied for both SYBR Green I and TaqMan assays. PCR reaction (40 cycles) was performed on an ABI Prism 7900HT Sequence Detection System (Applied Biosystems): initial denaturation for 10 minutes at 95°C, followed by 40 amplification cycles at 95°C (15 seconds), annealing at 60°C (1 min). To confirm product specificity melting curves were performed to verify that only the specific product was synthesized and to exclude the formation of primer dimers. The specificity of the PCR was further verified by subjecting the amplification products to agarose gel electrophoresis. Plasmids containing ADAM10, ADAM17 or β-actin were applied to produce standard curves which were used to calculate the exact copy number of each mRNA. Therefore the plasmid DNA was diluted in 10-fold serial dilutions (10^7^–10^2^ copies) and amplified along with cDNA samples. The copy numbers of the specific target genes were normalized to 10^6^ copies of the housekeeping gene β-actin.

### Immunodetection

After incubation with the respective substances the transfected cells were washed twice with PBS and were lysed in the respective lysis buffer. Equal amounts of protein were subjected to SDS-PAGE under reducing (cleaved caspase-3, cleaved PARP, ADAM10, pSTAT3, PDI, CHOP and BiP) or non reducing (ADAM17) conditions using Tris-Glycine gels (7.5–15%). The proteins were then transferred onto PVDF (Polyvinylidene Fluoride) (ADAM10 and ADAM17) or nitrocellulose membranes (cleaved caspase-3, cleaved PARP, pSTAT3, PDI, CHOP and BiP). After the transfer the membranes were blocked in 5% non-fat dried milk powder in PBS, 0.3% Tween20 and 0.05% Triton X-100 and further incubated with the respective primary antibody (1∶1,000–3,000) in PBS containing 1–5% (w/v) non-fat dried milk powder or 5% (w/v) BSA, 0.3% Tween and 0.05 % Triton X-100 at 4°C overnight. Anti-cleaved caspase-3, anti-cleaved PARP, anti-BiP, anti-PDI (Cell Signaling Technology, New England Biolabs, Frankfurt, Germany), anti-CHOP (Santa Cruz Biotechnology, Heidelberg, Germany), anti-ADAM10 (Sigma Aldrich, Munich, Germany), anti-ADAM17 (R&D Systems, Minneapolis, MN) and anti-GAPDH (Fitzgerald, Concord, MA) were used as primary antibodies. For detection of P-STAT3 and total STAT3 PathScan P-STAT3 (Tyr705), PathScan total STAT3 ELISA (Cell Signaling Technology) and PhosphoPlus STAT3 (Tyr705) Antibody Kit were used according to the manufacturer's instructions.

### ^125^I-leptin binding assay

The culture medium was aspirated and the cells were incubated in 1 ml Krebs-Ringer buffer supplemented with 0.5% BSA (w/v) per 6-well for 30 min at 37°C and 5% CO_2_. Subsequently 0.5 kBq leptin tracer (Mediagnost, Reutlingen, Germany) were added per well and the cells were incubated for 1 h at 37°C and 5% CO_2_ in the presence or absence of 3 µg/ml unlabeled leptin. Cells were washed twice with PBS/0.5% BSA (w/v) and lysed with 1 ml 0.1 M NaOH solution. Activity in the cell lysates was determined by gamma counter measurements (Berthold LB2111, Berthold Technologies, Bad Wildbad, Germany). Specific binding was determined by subtracting the radioactivity bound in the presence of 3 µg/ml unlabeled leptin (nonspecific binding) from the radioactivity bound in the absence of 3 µg/ml unlabeled leptin.

### Detection of sOb-R

sOb-R in the supernatant of transfected cells was detected with an in-house immunofunctional assay as described previously [15]. The in-house immunofunctional assay was also used for determination of Ob-R protein expression in whole cell lysates. The cell lysates were diluted 1∶5 in PBS before they were applied in the assay.

### Statistical analysis

Data are presented as means ± SD. For statistical analysis, we used the STATISTICA 6.0 software program. In the case of non-normally distributed data, non-parametric statistical tests were used for all statistical analyses. Otherwise parametric tests were applied. A P-value <0.05, was considered statistically significant.

## Results

### Constitutive Ob-R shedding and mediating proteases

#### Human membrane-anchored Ob-R isoforms are substrates for constitutive Ob-R cleavage

**Figure 1:**
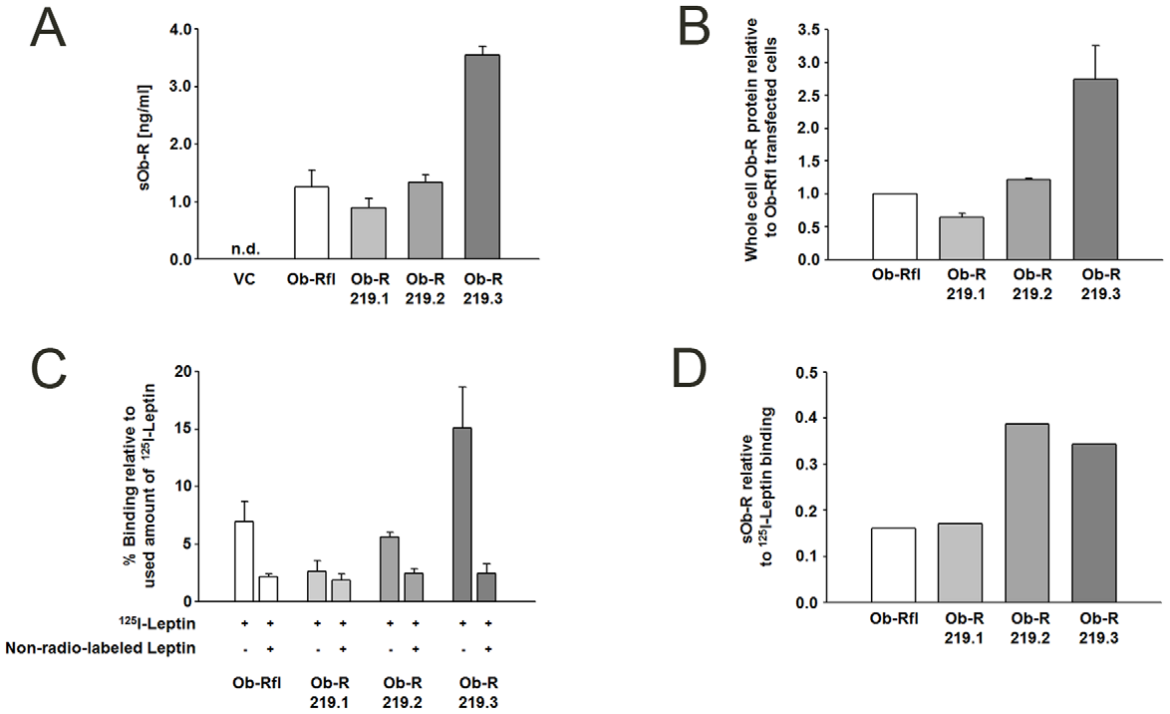
Constitutive shedding of human Ob-R isoforms. (A) sOb-R concentration in the supernatant of Ob-R transfected HEK cells after incubation for 48 h under normal culture conditions (37°C, 5% CO_2_). VC, vector control (pcDNA 3.1) (B) Ob-R in whole cell lysates of Ob-R transfected HEK cells was determined by an in-house immunofunctional assay. Ob-R protein expression is presented realtive to Ob-R levels of Ob-Rfl transfected cells. (C) I^125^-leptin binding of Ob-R transfected HEK cells. Ob-R transfected cells were incubated for 48 h under normal culture conditions (37°C, 5% CO_2_) and I^125^-leptin binding assay was performed. I^125^-leptin binding is shown in % relative to amount of used I^125^-leptin tracer. (D) Normalisation of sOb-R (A) to I^125^-leptin binding (C). Data are presented as means ± SD of n≥3 experiments; n.d., not detectable.

Although it is known that sOb-R is generated through cleavage of membrane-anchored receptors, is it not yet clear which isoforms serve as substrate and which protease(s) mediate(s) the cleavage process. So we transfected HEK cells with the long (Ob-Rfl) or one of the three short Ob-R isoforms (Ob-R 219.1, Ob-R 219.2 and Ob-R 219.3) to determine if sOb-R is constitutively generated from these four receptor isoforms. After 48 h incubation at 5% CO_2_ and 37°C the cell supernatants were collected. Fig. 1A shows that all isoforms could serve as substrate for the shedding process. ^125^I-leptin binding assays and determination of Ob-R protein expression in whole cell lysates were performed as expression and functionality control for the different Ob-R isoforms. Fig. 1 B and C illustrate that short Ob-R isoforms 219.2 and 219.3 showed the highest Ob-R protein-expression in whole cell lysates as well as the highest leptin binding. When the amount of generated sOb-R was normalized to the membrane expression of the respective isoform, determined by ^125^I-leptin binding, there was a trend for an inverse correlation between the length of the cytoplasmic domain and the amount of shed Ob-R (Fig. 1 D). Similar results were obtained through normalization of sOb-R to Ob-R protein in whole cell lysates of HEK cells transfected with the respective Ob-R isoforms (Supporting information Fig. S1). Ob-Rfl was demonstrated to be the main signal-transducing isoform [11] and Ob-R219.3 showed the highest shedding rate in our *in-vitro* experiments. Therefore, we focused further investigations in the present study on these two Ob-R-isoforms.

#### ADAM10 predominantly mediates constitutive Ob-R shedding

**Figure 2:**
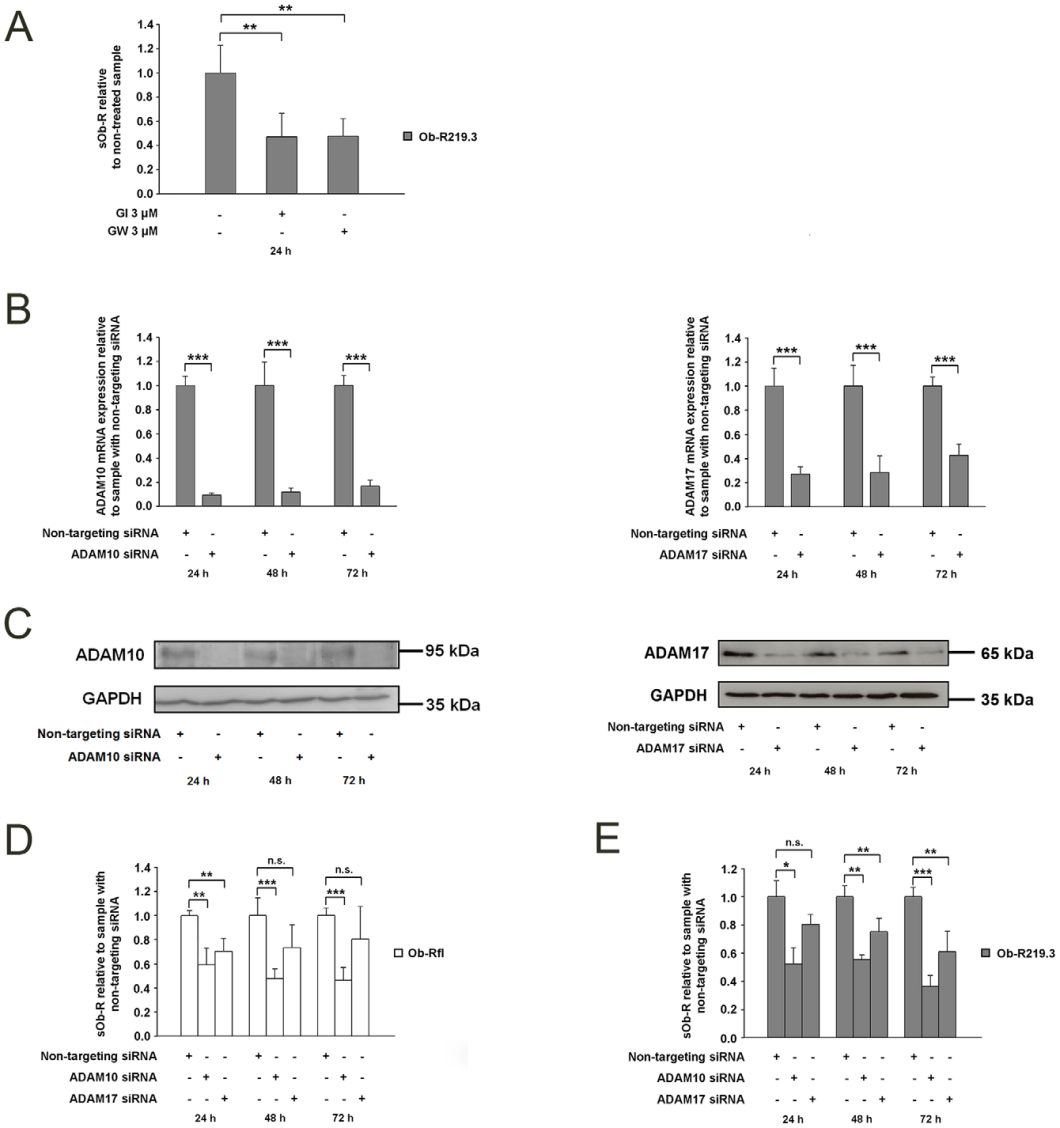
Constitutive shedding of human Ob-R isoforms is mainly mediated by ADAM10. (A) sOb-R levels in the supernatant of Ob-R219.3 transfected HEK cells after incubation with the metalloprotease inhibitors GI/GW (3 µM) or DMSO (0.1%) for 24 h. sOb-R levels of GI/GW incubated cells are presented relative to those of cells incubated with DMSO. (B and C) ADAM10 and ADAM17 mRNA (B) and protein expression (C) in lysates of HEK cells transfected with a specific siRNA for ADAM10 or ADAM17 or a non-targeting siRNA (50 nM). (D and E) sOb-R levels in the supernatant of HEK cells transfected with a specific siRNA for ADAM10, ADAM17 or a non-targeting siRNA (50 nM) and subsequently with Ob-Rfl (D) or Ob-R219.3 (E) followed by a further incubation under normal culture conditions for 24–72 h. sOb-R levels of ADAM10/ADAM17 siRNA transfected cells are shown relative to those of cells transfected with a non-targeting siRNA. Data are presented as means ± SD of n≥3 experiments.

The possible involvement of metalloproteases in constitutive Ob-R shedding was investigated by use of the broad spectrum metalloprotease inhibitor GM6001. The incubation of Ob-R219.3 transfected cells with GM6001 (25–100 µM) for 24 h and 48 h (Supporting information Fig. S2) decreased shedding by up to 52% (P<0.01). Metalloproteases ADAM10 and ADAM17 are probably the best characterized members of the ADAM family and they are known to catalyze shedding of membrane proteins which show structural similarities to Ob-R such as the interleukin 15 receptor alpha (IL-15Rα), the interleukin 6 receptor (IL-6R) and the growth hormone receptor [37]–[39]. To investigate the effect of ADAM10 and ADAM17 on Ob-R shedding, Ob-R219.3 transfected HEK cells were incubated with either one of two metalloprotease inhibitors: GI254023X (GI) that blocks ADAM10 with a 100-fold higher specificity towards ADAM10 than to other proteases or GW280264X (GW) that blocks both ADAM10 and ADAM17 with comparable IC50 values [40], [41]. Fig. 2 A reveals that GI diminished constitutive shedding by 53% (P<0.01) whereas incubation with GW resulted in 52% (P<0.01) decreased sOb-R levels. To further verify to which extent the different ADAMs participate in Ob-R cleavage of the two selected isoforms, we next performed ADAM10 and ADAM17 siRNA knockdown experiments. HEK cells were first transfected with an ADAM10 or ADAM17 specific siRNA or a non-targeting siRNA. 24 hours later the siRNA transfected cells were transfected with Ob-Rfl or Ob-R219.3. Quantitative RT-PCR measurements revealed a knockdown of ADAM10 or ADAM17 mRNA expression of at least 60% (P<0.001) 24 h–72 h post transfection (Fig. 2 B). The observed mRNA knockdown was paralleled by a decrease in ADAM10 and ADAM17 protein levels (Fig. 2 C). ADAM10 knockdown sufficiently blocked the constitutive release of sOb-R into the supernatant of Ob-Rfl transfected cells by up to 57% (Fig. 2 D, P<0.001) whereas ADAM17 knockdown less effectively diminished sOb-R levels up to 30% (P<0.01). The release of sOb-R by Ob-R219.3 transfected cells was decreased with a maximum of 64% (Fig. 2 E P<0.001) after ADAM10 but only up to 39% (P<0.01) by ADAM17 siRNA-mediated knockdown (Fig. 2 E).

### Induction of Ob-R shedding

#### PKC stimulation increases Ob-R shedding

**Figure 3:**
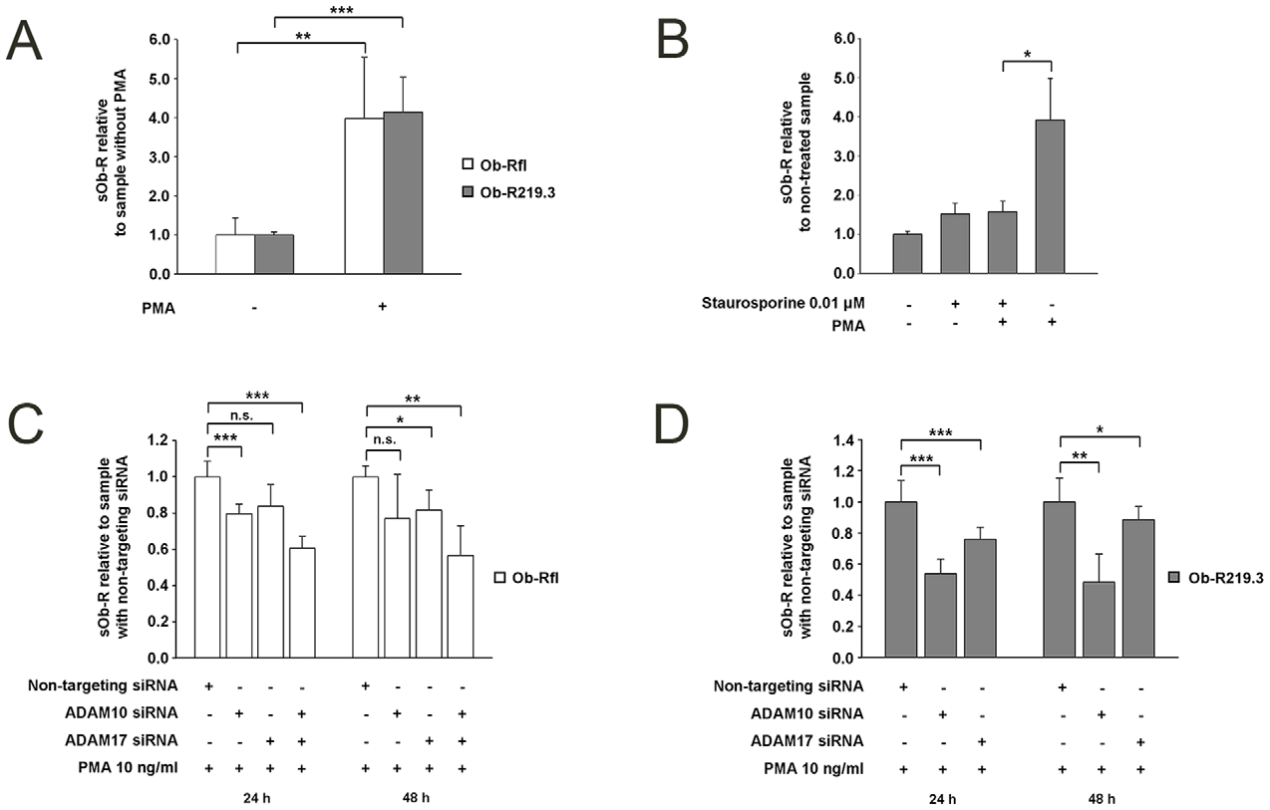
PKC activation leads to an increased release of sOb-R by Ob-R transfected cells, mainly mediated by ADAM10. (A) sOb-R levels in the supernatant of Ob-Rfl or Ob-R219.3 transfected cells after incubation with PMA (10 ng/ml) or DMSO (0.1%) for 24 h. sOb-R levels of PMA-treated cells are displayed relative to sOb-R levels of DMSO-treated cells. (B) sOb-R levels in the supernatant of Ob-R219.3 transfected cells after incubation with PMA (10 ng/ml) and or staurosporine (0.01 µM) or DMSO (0.1%) for 24 h. sOb-R levels of PMA- and staurosporine-treated cells are shown relative to those of cells treated with DMSO. (C, D) sOb-R levels in the supernatant of HEK cells transfected with a specific siRNA for ADAM10 and or ADAM 17 or a non-targeting siRNA (50 nM) and subsequently with Ob-Rfl (C) or Ob-R219.3 (D) followed by a further incubation for 24–72 h with PMA (10 ng/ml). sOb-R levels of ADAM10/ADAM17 siRNA transfected cells are presented relative to sOb-R levels of cells transfected with a non-targeting siRNA. Data are presented as means ± SD of n≥3 experiments.

It is known that metalloprotease-mediated shedding processes can be induced by activation of PKC [42]. Hence we transfected HEK cells transiently with Ob-Rfl or Ob-R219.3 and stimulated these cells with the PKC-activator PMA (10 ng/ml) for 24 h. The incubation resulted in a 3.98-fold (P<0.01)/4.14-fold (P<0.001) increase of sOb-R in the cell supernatant (Fig. 3 A). Co-incubation of Ob-R219.3 transfected cells with the protein kinase inhibitor staurosporine (0.01 µM) inhibited the PMA stimulated increase of sOb-R concentration after 24 h by 60% (P<0.05) (Fig. 3 B).

#### PKC-induced Ob-R shedding is mediated by ADAM10 and to a minor degree by ADAM17

To elucidate the importance of ADAM10 and ADAM17 for PKC-induced Ob-R shedding, Ob-R219.3 transfected cells were incubated with the specific ADAM10 or ADAM10/17 inhibitors GI and GW for 24 h. Results shown in supporting information Fig. S3 reveal that GI diminished PMA-induced shedding by 48% (P<0.01) and that GW-mediated decrease was 51% (P<0.01). To clarify the effect of either ADAM on PKC-induced Ob-R shedding, we knocked down ADAM10 and ADAM17 in Ob-Rfl or Ob-R219.3 transfected HEK cells. PMA-induced shedding of Ob-Rfl transfected cells was only decreased by up to 20% (P<0.05) independently of the siRNA applied (Fig. 3 C). However simultaneous knockdown of ADAM10 and ADAM17 led to a significant decrease of 43% (P<0.01) supporting that both ADAMs participate in PKC-induced shedding of Ob-Rfl. PMA-induced elevation of sOb-R in the supernatant of Ob-R219.3 transfected cells was dimished by 52% (P<0.01) after knocking down ADAM10 (Fig. 3 D) compared to the sample treated with non-targeting siRNA. Knockdown of ADAM17 only decreased sOb-R concentration by 24% (P<0.001).

#### Caspase-mediated apoptosis increases Ob-R shedding dependent on ADAM10 expression

**Figure 4:**
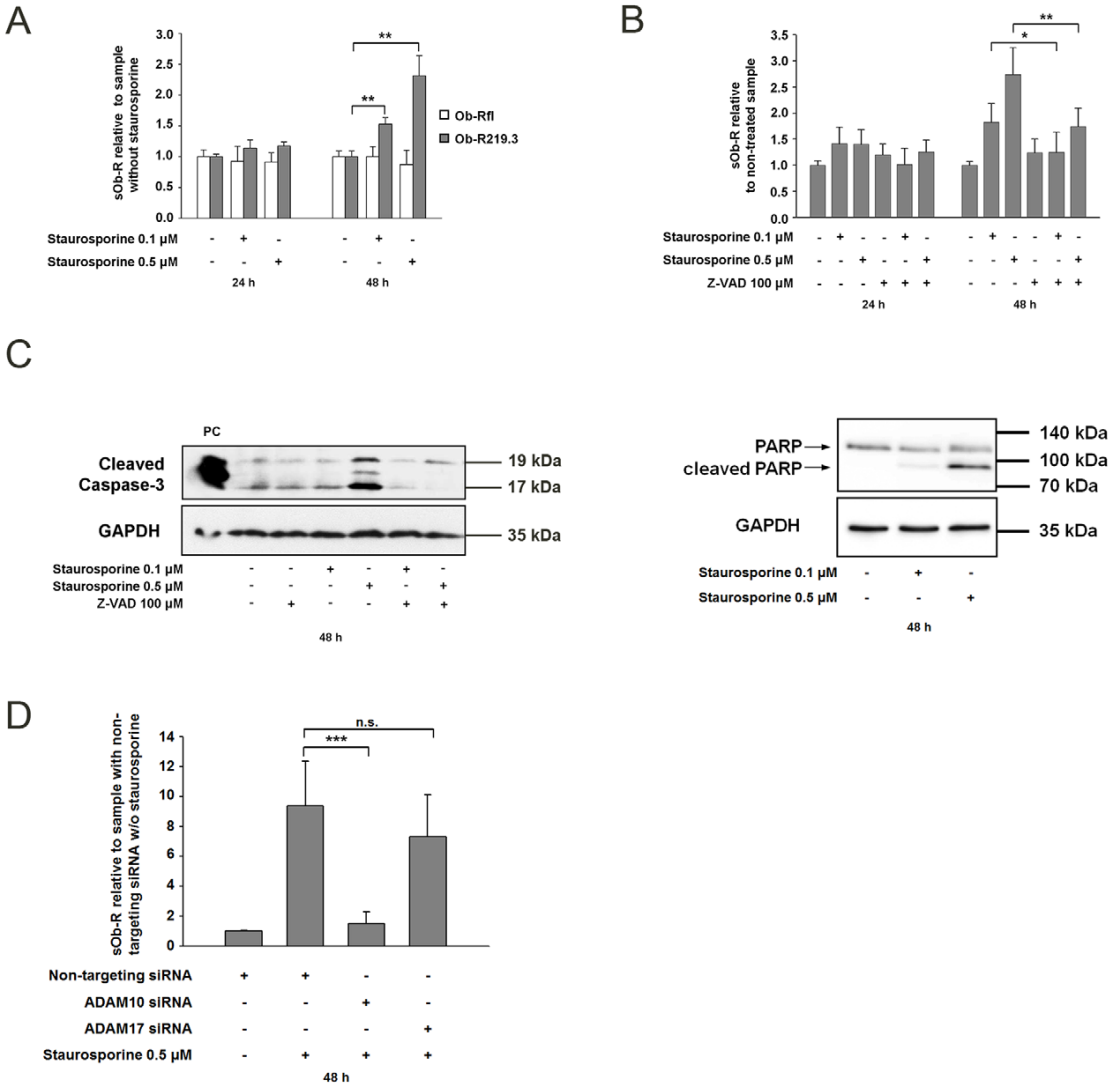
Caspase-mediated apoptosis, induced by incubation with staurosporine, activates Ob-R shedding mainly mediated by ADAM10. (A) sOb-R levels in the supernatant of Ob-Rfl or Ob-R219.3 transfected cells after incubation with staurosporine (0.1–0.5 µM) or DMSO (0.1%) for 24 h and 48 h. sOb-R levels of staurosporine-treated cells are displayed relative to sOb-R levels of cells treated with DMSO (B) sOb-R levels in the supernatant of Ob-R219.3 transfected cells after pre-incubation with the broad spectrum caspase inhibitor Z-VAD(OMe)FMK (100 µM) or DMSO (0.1%) for 30 min and subsequent stimulation with staurosporine (0.1–0.5 µM) or DMSO (0.1%) for 24 h and 48 h. sOb-R levels of staurosporine- or Z-VAD(OMe)FMK-treated cells are shown relative to those of cells treated with DMSO. (C) Representative western blot of cleaved caspase-3 and cleaved PARP after staurosporine incubation. Ob-R219.3 transfected cells were pre-incubated with Z-VAD(OMe)FMK (100 µM) or DMSO (0.1%) for 30 min and subsequently stimulated with staurosporine (0.1–0.5 µM) or DMSO (0.1%) for 48 h. Following this incubation cleaved caspase-3 and cleaved PARP in cell lysates was determined by western blot analysis. PC, positive control (cell lysate of Jurkat cells treated with cytochrome c). (D) sOb-R levels in the supernatant of HEK cells transfected with a non-targeting, ADAM10 or ADAM17 specific siRNA (50 nM) and subsequently with Ob-R219.3 after incubation with staurosporine (0.5 µM) or DMSO (0.1%) for 48 h. sOb-R levels of ADAM10 and ADAM17 siRNA transfected cells are presented relative to sOb-R levels of cells transfected with a non-targeting siRNA and treated with DMSO. Data are presented as means ± SD of n≥3 experiments.

Staurosporine, beside its inhibitory effect on protein kinases is known to be an effective inducer of apoptosis. To investigate a potential association between apoptosis and Ob-R shedding we incubated Ob-R219.3 transfected cells with staurosporine (0.1–0.5 µM) for 24 h and 48 h. This resulted in up to 2.31-fold (P<0.01) increased sOb-R levels compared to the non-treated control (Fig. 4 A) after 48 h. When investigating the long isoform Ob-Rfl the level of sOb-R remained comparable to control data (Fig. 4 A). Western blots for cleaved caspase-3 and cleaved Poly-(ADP-ribose) Polymerase (PARP) (Fig. 4C) and ELISA measurements of nucleosome release into the cytoplasm (Supporting information Fig. S4) proved successful induction of apoptosis by 0.5 µM staurosporine after 48 h. Pre-incubation with broad spectrum caspase inhibitor Z-VAD(OMe)-FMK(100 µM) impaired induction of apoptosis and diminished staurosporine-mediated increase of sOb-R by up to 3%(P<0.05) (Fig. 4 B and C). To clarify if ADAM10 and ADAM17 are involved in staurosporine-induced shedding we knocked down ADAM10 or ADAM17 with a specific siRNA as described before and incubated Ob-R219.3 transfected cells with staurosporine 0.5 µM for 48 h. Fig. 4D shows that ADAM10 siRNA knockdown resulted in 84% (P<0.01) whereas a knockdown of ADAM17 diminished sOb-R levels by 23% (p = 0.26) compared to the sample transfected with a non-targeting siRNA. To elucidate if the effects on sOb-R generation were staurosporine-specific we incubated Ob-R transfected HEK cells with doxorubicin (inhibitor of topoisomerase II) as a more specific inducer of apoptosis. We demonstrated that doxorubicin increased the amount of shed Ob-R219.3 in a comparable manner as 0.1 µM Staurosporine, whereas no significant changes could be determined for Ob-Rfl (Supporting information Fig. S5).

#### **Lipotoxicity and apoptosis induced by the saturated FFA palmitate increase sOb-R levels**

**Figure 5:**
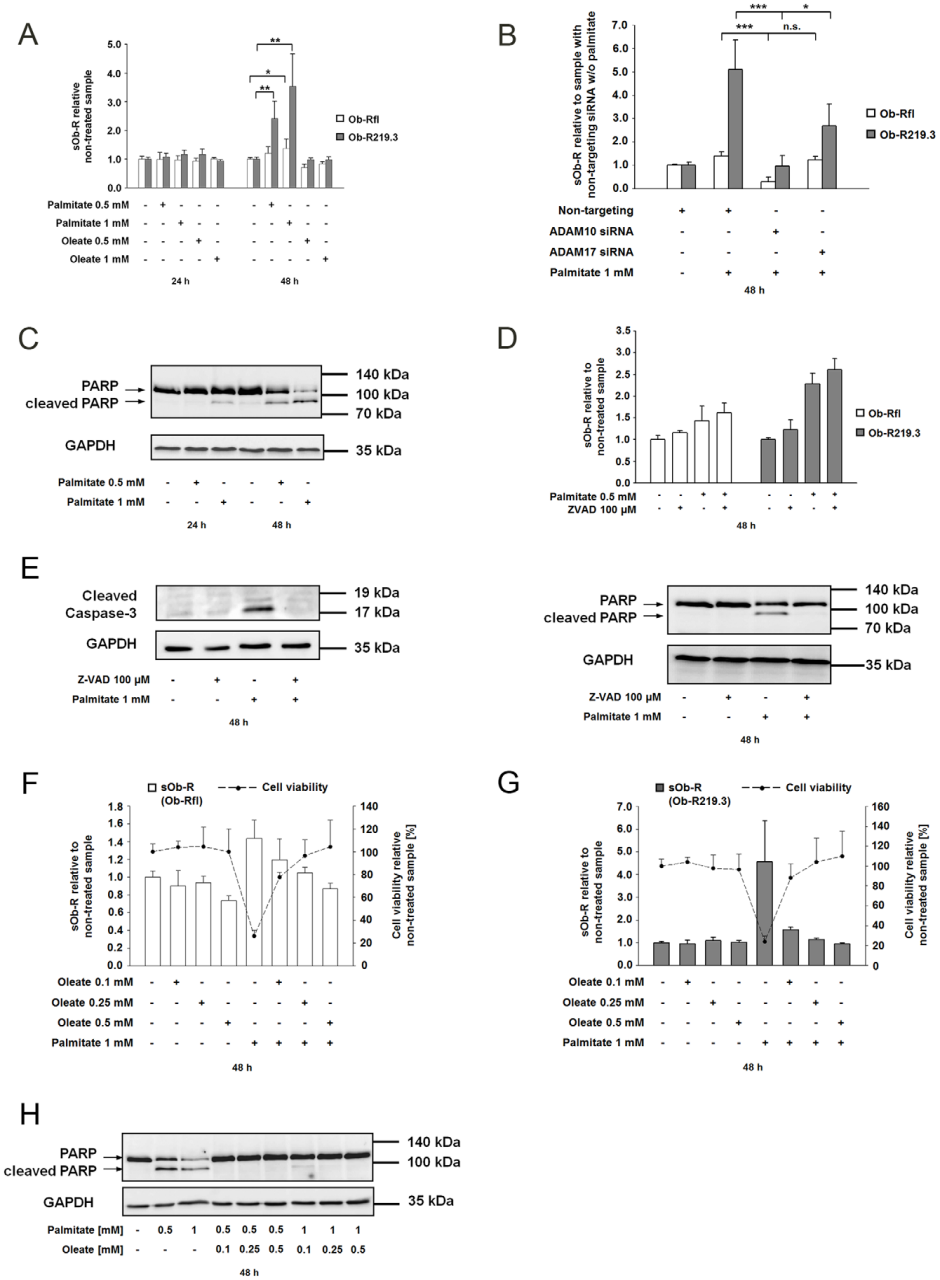
Enhanced release of sOb-R after palmitate incubation is mediated by ADAM10 and ADAM17 and inhibited by oleate. (A) sOb-R levels in the supernatant of Ob-Rfl or Ob-R219.3 transfected cells after incubation with palmitate (0.5–1 mM), oleate (0.5 mM–1 mM or methanol (2%) for 24 and or 48 h. sOb-R levels of palmitate- or oleate-treated cells are displayed relative to those of cells treated with methanol. (B) sOb-R levels in the supernatant of HEK cells transfected with a non-targeting, ADAM10 or ADAM17 specific siRNA (50 nM) and subsequently with Ob-R219.3, after incubation with palmitate (1 mM) or methanol (2%) for 48 h. sOb-R levels of ADAM10 and ADAM17 siRNA transfected cells are shown relative to sOb-R levels of cells transfected with a non-targeting siRNA and treated with methanol. (C) Representative western blot of cleaved PARP after palmitate incubation. Ob-R transfected cells were incubated with palmitate (0.5–1 mM) or Methanol (2%) for 24 and 48 h. Following this incubation cleaved PARP in cell lysates was determined by western blot analysis. (D) sOb-R levels in the supernatant of Ob-Rfl or Ob-R219.3 transfected cells after incubation with palmitate 1 mM and Z-VAD 100 µM or methanol (2%) for 48 h. sOb-R levels of palmitate- and or Z-VAD-stimulated cells are displayed relative to those of cells treated with methanol. (E) Representative western blot of cleaved caspase-3 and cleaved PARP. Ob-R transfected cells were incubated with palmitate (0.5–1 mM), palmitate (0.5–1 mM) and Z-VAD (100 µM) or Methanol (2%) for 48 h. Following this incubation cleaved caspase-3 and cleaved PARP in cell lysates was determined by western blot analysis. (F and G) sOb-R levels in the supernatant of Ob-Rfl or Ob-R219.3 transfected cells after incubation with palmitate (1 mM), oleate (0.1–0.5 mM), palmitate (1 mM) and oleate (0.1–0.5 mM) or methanol (2%) for 48 h. sOb-R levels of palmitate-, oleate– or palmitate- and oleate-treated cells are displayed relative to those of cells treated with methanol. (H) Representative western blot of cleaved PARP after co-incubation with palmitate and oleate. Ob-R transfected cells were incubated with palmitate (0.5–1 mM), palmitate (0.5–1 mM) and oleate (0.1–0.5 mM) or Methanol (2 %) for 48 h. Following this incubation cleaved PARP in cell lysates was determined by western Blot analysis. Data are presented as means ± SD of n≥3 experiments (Fig. 5 D and E, n = 2).

Lipotoxicity caused by elevated free fatty acids in tissues other than the adipose tissue could finally cause cell death through apoptosis. So we speculated that free fatty acids influence Ob-R cleaveage. By incubating Ob-R219.3 or Ob-Rfl transfected HEK cells with oleate or palmitate (0.5 mM–1 mM) we could show that palmitate but not oleate increased the amount of shed Ob-R. Ob-R219.3 transfected cells showed up to 3.5-fold (P<0.01) and Ob-Rfl transfected cells up to 1.4-fold(P<0.05) elevated sOb-R levels in the supernatant after 48 h (Fig. 5 A). To elucidate the role of ADAM10 and ADAM17 in palmitate-mediated shedding we knocked down ADAM10 or ADAM17 with a specific siRNA as described before and incubated Ob-R219.3 or Ob-Rfl transfected cells with palmitate 1 mM for 48 h. Fig. 5 B displays that knockdown of ADAM10 resulted in up to 81% (Ob-R219.3, P<0.01) or 87% (Ob-Rfl, P<0.05) decreased amounts of sOb-R whereas a knockdown of ADAM17 diminished sOb-R levels by 44% (Ob-R219.3, P<0.05) or 11% (Ob-Rfl, P = 0.11) compared to cells treated with a non-targeting siRNA. The observed increase was accompanied by decreased cell viability measured by CCK-8 assay (data not shown) as a consequence of apoptosis demonstrated through detection of cleaved caspase-3 and cleaved PARP (Fig. 5 C and E). Interestingly a co-incubation of Ob-R-transfected cells with palmitate 1 mM and broad spectrum caspase inhibitor Z-VAD(OMe)-FMK (100 µM) inhibited induction of caspase-3 and PARP-cleavage but did neither restore cell viability (data not shown) nor decreased sOb-R to basal levels (Fig. 5 D and E). In contrast co-incubation of Ob-R transfected cells with palmitate 0.5–1 mM and increasing concentrations of oleate 0.1–0.5 mM reversed the lipotoxic effects of palmitate (Fig. 5F–H) and decereased sOb-R concentration to basal levels (Fig. 5F and G).

### Modulation of leptin-mediated STAT3 phosphorylation by sOb-R and Ob-R isoform 219.3

**Figure 6:**
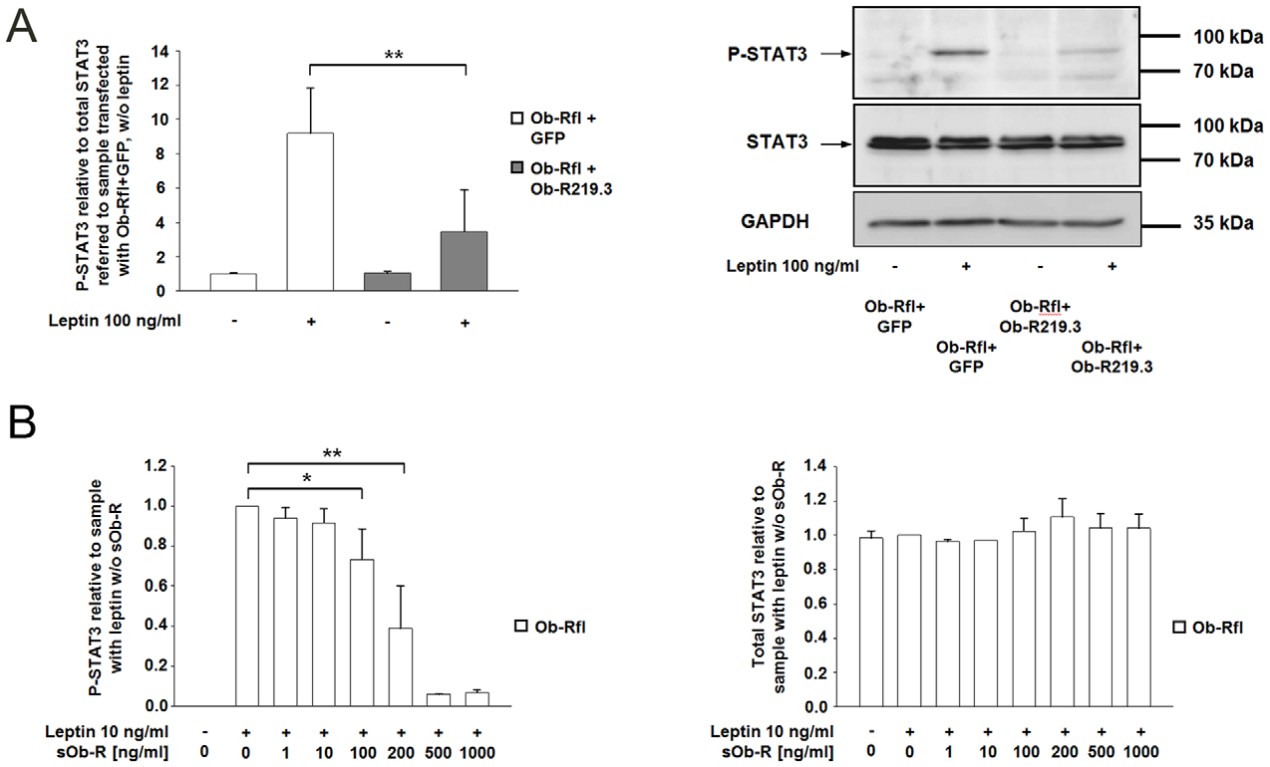
Co-expression of Ob-R219.3 to Ob-Rfl and increased sOb-R levels modulate leptin signaling of Ob-Rfl. (A) Ob-R transfected cells were serum-starved for 16 h and subsequently incubated with leptin (100 ng/ml) for 30 min. P-STAT3 and total STAT3 levels in lysates of HEK cells transfected with equimolar amounts of Ob-Rfl + GFP (equimolar to Ob-R219.3) or Ob-Rfl + Ob-R219.3 were determined by ELISA and western blot. P-STAT3/total STAT3 ratios are displayed relative to P-STAT3/total STAT3 ratio of non-treated cells transfected with Ob-Rfl + GFP. (B) P-STAT3 and total STAT3 levels in lysates of HEK cells transfected with Ob-Rfl. Ob-Rfl-transfected cells were stimulated with media containing leptin (10 ng/ml) and increasing concentrations of sOb-R (0–1000 ng/ml). P-STAT3 and total STAT3 levels are displayed relative to those of leptin-treated control without sOb-R. Data are presented as means ± SD of n≥2 experiments.

We elucidated if leptin actions via STAT3 phosphorylation might be modulated by co-expression of Ob-R219.3. Ob-Rfl and Ob-R219.3 or Ob-Rfl and GFP (vector control, equimolar to Ob-R219.3) co-transfected cells were serum starved for 16 h and afterwards stimulated with leptin (100 ng/ml) for 30 min. Fig. 6A illustrates that the co-expression of Ob-Rfl and Ob-R219.3 reduced leptin-induced STAT3 activation by 62.2% (P<0.01)compared to cells transfected with Ob-Rfl + GFP only. To clarify the influence of increasing sOb-R levels on leptin signaling we pre-incubated medium containing 10 ng/ml leptin with sOb-R (1–1000 ng/ml) for one hour at 37°C. Afterwards the medium was applied to stimulate 16 h serum-starved HEK cells for 30 min. Fig. 6 B shows that increasing sOb-R concentrations reduced leptin-mediated STAT3 phosphorylation compared to Ob-Rfl transfected cells incubated with medium free of sOb-R. An effect of 20–94% inhibition was detectable for the addition of 100–1000 ng/ml sOb-R, whereas total STAT3 levels remained unchanged.

### sObR generation is regulated by leptin level and ER stress

#### Leptin decreases sOb-R concentrations

**Figure 7:**
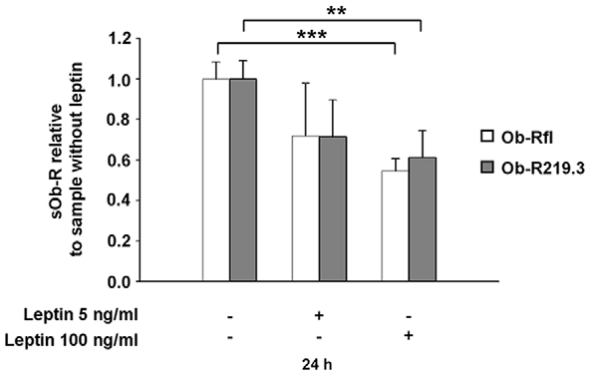
Leptin inhibits sOb-R release from Ob-Rfl or Ob-R219.3 transfected cells into the supernatant. Ob-Rfl or Ob-R219.3 transfected cells were incubated with leptin (5–100 ng/ml) for 24 h. Following this incubation sOb-R in the supernatant was determined. sOb-R levels of leptin-treated cells are shown relative to those of non-treated cells. Data are presented as means ± SD of n≥3 experiments.

In adiposity high leptin levels are correlated with decreased sOb-R levels [27]–[29]. As such a leptin excess could result in an Ob-R downregulation we asked, whether this process leads to a decline in the constitutive generation of sOb-R. Therefore, we incubated Ob-Rfl or Ob-R219.3 transfected HEK cells for 24 h with 5–100 ng/ml leptin. Fig. 7 demonstrates that 100 ng/ml leptin decreased the amount of sOb-R in the cell supernatant by up to 46%(P<0.001) for Ob-Rfl and up to 39% (P<0.01) for Ob-R219.3. The decrease was concentration-dependent but independent of the length of the transfected Ob-R isoform.

#### ER stress decreases membrane Ob-R expression, impairs leptin signaling and reduces sOb-R generation

**Figure 8:**
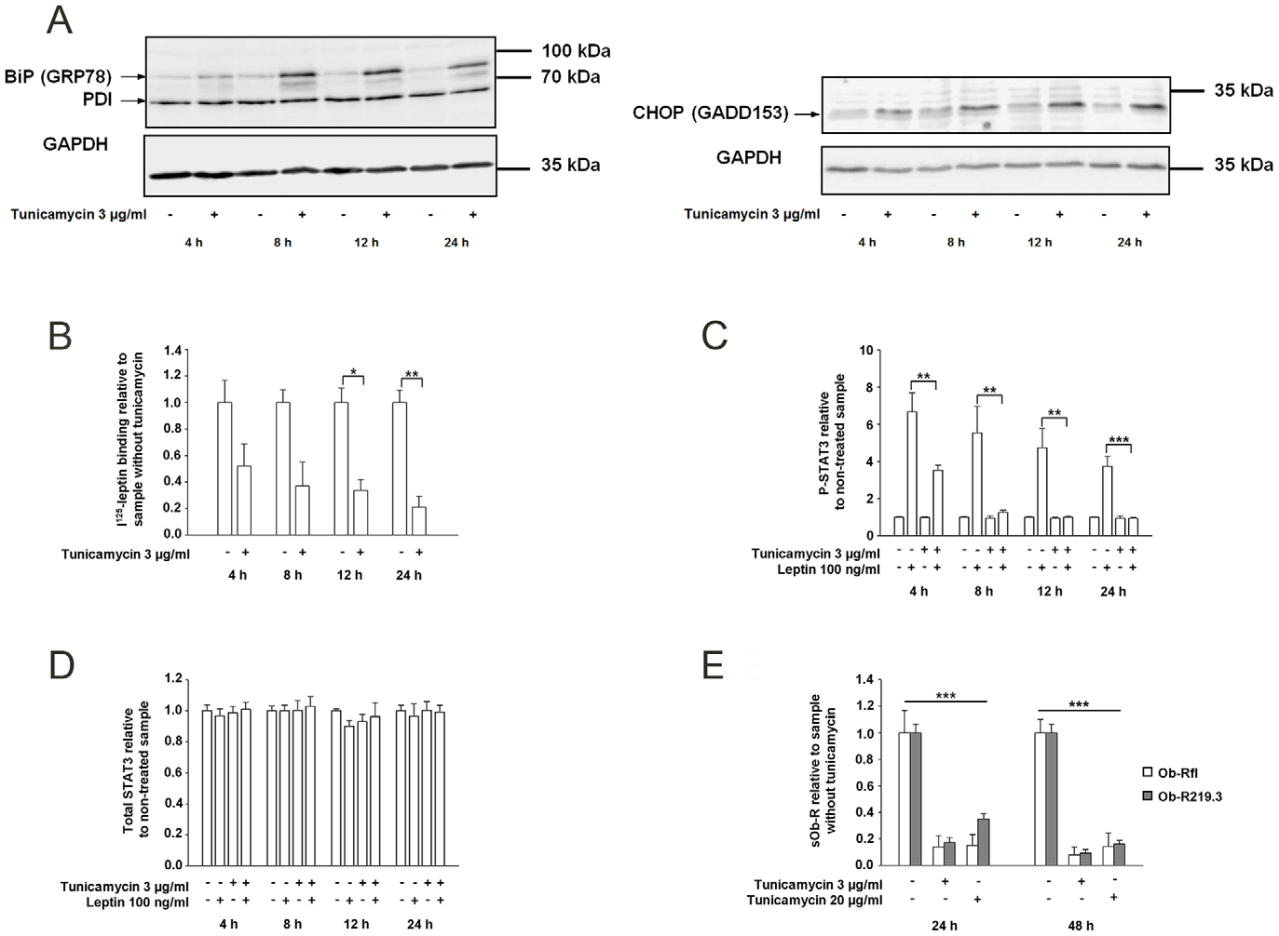
ER stress causes downregulation of membrane Ob-R, impaired leptin signaling via Ob-Rfl and decreased sOb-R concentrations. (A) Representative western blot for protein expression of ER stress markers BiP (GRP78), CHOP (GADD153) and PDI after treatment of Ob-R transfected HEK cells with tunicamycin (3 µg/ml) or DMSO (0.1%) for 4–24 h. (B) Ob-Rfl transfected cells were incubated with tunicamycin (3 µg/ml) or DMSO (0.1%) for 4–24 h and I^125^-leptin binding assay was performed. I^125^-leptin binding of tunicamycin-treated samples is displayed relative to that of non-treated controls. (C and D) P-STAT3 (C) and total STAT3 (D) levels in lysates of Ob-Rfl transfected cells after incubation with tunicamycin (3 µg/ml) or DMSO (0.1%) for 4–24 h and subsequent stimulation with leptin (100 ng/ml) for 30 min. P-STAT3 and total STAT3 levels of leptin-stimulated cells are displayed relative to that of non-treated controls. (E) sOb-R levels of Ob-Rfl or Ob-R219.3 transfected cells after incubation with tunicamycin (3–20 µg/ml) or DMSO (0.2%) for 24 and 48 h. sOb-R levels of tunicamycin-treated cells are displayed relative to those of non-treated cells. Data are presented as means ± SD of n≥3 experiments (Fig. 8 A, n = 1; Fig. 8 B, n = 2).

Obesity is a state of low grade inflammation associated with ER stress. To investigate whether or not membrane Ob-R expression and leptin signaling are affected by ER stress we applied tunicamycin, an inhibitor of protein N-glycosylation. Ob-Rfl transfected cells were incubated with tunicamycin for 4–24 h and subsequently stimulated with leptin (100 ng/ml) for 30 min. Cells and cell lysates were subjected to ^125^I-leptin binding assay, P- and total STAT3 ELISA and determination of ER stress markers, respectively. Cell viability did not change significantly over the 24 h of tunicamycin incubation determined by CCK-8 assay (data not shown). Fig. 8A illustrates the successful activation of ER-stress markers “immunoglobulin heavy chain binding protein” (BiP), also referred as 78-kDa glucose-regulated protein (GRP78) and the C/EBP homology protein (CHOP). Another marker protein named protein-disulfide-isomerase (PDI) remained unaffected. ^125^I-leptin binding assay revealed a 60% decreased leptin binding after 4 h of tunicamycin incubation which further declined to 15% of control level after 24 h (Fig. 8 B). Leptin-induced STAT3 phosphorylation was decreased by 47% (P<0.01) after 4 h and completely blocked after 12 h incubation with tunicamycin (Fig. 8 C). Total STAT3 amounts remained unchanged after tunicamycin treatment (Fig. 8 D). To elucidate if the reduced membrane Ob-R expression is reflected by decreased sOb-R levels, we incubated Ob-Rfl as well as Ob-R219.3 transfected cells with tunicamycin (3–20 µg/ml) for 24–48 h. Fig. 8E shows that tunicamycin incubation led to 65% (P<0.001) decreased sOb-R levels compared to the non-treated sample already after 24 h. The inhibition reached a maximum of 92% and 90% for Ob-Rfl and Ob-R219.3 after 48 h.

## Discussion

In previous studies we and others showed that sOb-R represents the main leptin binding activity in human blood [15] and modulates the bioavailability of free leptin [15]–[19]. Detailed knowledge about the basic shedding mechanisms of Ob-R is mandatory to manipulate leptin actions by increasing or decreasing sOb-R levels in conditions of leptin deficiency or resistance. In the present study we elucidated regulatory mechanisms for sOb-R generation covering receptor shedding and receptor trafficking in HEK cells with a transient overexpression of different Ob-R isoforms. We evidenced that the four known human membrane-anchored Ob-R isoforms were substrates for generation of sOb-R with constitutively low levels. The degree to which each isoform contributes to the pool of serum sOb-R appeared to correlate with the respective Ob-R total and membrane protein expression and seemed to be inversely associated with the length of the cytoplasmic domain. Previously, Ob-Rfl was demonstrated to be the main signal-transducing isoform [11] and Ob-R219.3 was shown to be expressed abundantly in peripheral tissues and displayed the highest shedding rate in our *in-vitro* experiments. Therefore, we focused our investigations in the present study on these two physiologically relevant Ob-R-splice variants. ADAM10 and or ADAM17 are known members of the metalloprotease family that cleave transmembrane receptors like IL-6R, IL-15Rα and the growth hormone receptor which like Ob-R belong to the class I cytokine receptor family [37]–[39]. Downregulation of sOb-R by ADAM10 or ADAM17 inhibitors and through ADAM specific siRNA knockdown identified ADAM10 as the major protease for constitutive Ob-R shedding. ADAM17 appeared to be involved in the constitutive cleavage process only to a minor degree. This hypothesis was further supported by measurements of sOb-R levels in serum of ADAM17^ex/ex^ mice (data not shown) [43]. Serum sOb-R concentrations of these mice did not differ significantly from those of wild type mice despite of the protease knockdown. We further clarified mechanisms that induce or reduce the cleavage of membrane Ob-R. The detected increase of Ob-R shedding upon PKC activation is in line with previously published results that showed an activating effect of PKC stimulation on Ob-R cleavage [13], [14]. Interestingly, our data point to an isoform-dependent differential regulation of the shedding process: while ADAM10 appears to be responsible for PMA-induced shedding of Ob-R219.3, Ob-Rfl was shed by ADAM10 as well as ADAM17 in response to PMA treatment. As we could not completely inhibit constitutive and PKC-activated Ob-R shedding by knocking down ADAM10 and ADAM17 in our cell culture experiments, effects of other proteases cannot be fully excluded. Recently, Wauman et al. [44] showed that ADAM10 and ADAM17 were involved in RNF41 induced shedding of the human long Ob-R isoform and mouse short Ob-R isoform which confirms our findings that especially these two ADAM family members play an important role in activated Ob-R ectodomain-shedding. Additionally, we demonstrated that apoptosis induced by staurosporine and doxorubicin (Supporting information Fig. S5) activated shedding from Ob-R219.3 transfected cells. In contrast, the shedding rate of Ob-Rfl transfected cells remained negligible with both substances. This is interesting because it is in contrast to PMA incubation which increased shedding of Ob-Rfl and Ob-R219.3 to approximately the same extent. These results suggest that the amount of shed Ob-R depends on the shedding stimulus and the isoform-specific length of the cytoplasmic domain. Perhaps turnover of Ob-Rfl is greater than of Ob-R219.3, which would result in a bigger abundance of the short isoform Ob-R219.3 compared to the long isoform Ob-Rfl. A similar effect had been described for the human growth hormone receptor [45]. In addition, the longer intracellular domain of Ob-Rfl could also have a stabilizing effect and therefore protects Ob-Rfl from shedding. Differences in shedding efficacy between the short isoform Ob-R219.3 and the long isoform Ob-Rfl might be of importance for leptin actions. One supposed example for the physiological relevance of this process is leptin-transport across the blood brain barrier which is suggested to be dependent on the short isoforms [46], [47]. The preferential shedding of short isoforms could restrain this transport leading to impaired central leptin actions without affecting its peripheral effects. Moreover, in peripheral tissues with high expression of short Ob-R isoforms increased shedding could have a dual effect on leptin signaling via Ob-Rfl. Shedding of preferably short Ob-R isoforms could make the cells more sensitive to leptin. But with increasing levels of generated sOb-R an inhibitory effect of the released Ob-R ectodomain on leptin signaling may prevail. By use of the broad spectrum caspase inhibitor Z-VAD(OMe)-FMK we proved that increased shedding due to staurosporine treatment depends in part on the activation of caspases. Since the applied caspase inhibitor did not decrease the shedding rate to the basal level, a participation of caspase-independent mechanisms cannot be excluded. This hypothesis is further supported by the finding that staurosporine induces apoptosis via caspase-dependent as well as -independent ways [48]. Our finding that staurosporine-induced shedding of Ob-R mainly depends on the activity of ADAM10 is similar to mechanisms reported for distinct, even unrelated membrane receptors and proteins such as IL-6R, L-selectin and L1 [38], [49]–[52]. Therefore, ADAM-mediated cleavage of membrane proteins as consequence of induced apoptosis appears to be a general shedding mechanism. Because FFAs, particularly saturated, long chain fatty acids like palmitate or stearate, are also known to induce apoptosis [23], [24], we speculated that they could be major players for the induction of shedding as well. Fatty acid-induced shedding of both Ob-R isoforms that we observed in our investigations was mediated primarily by ADAM10 with a minor participation of ADAM17 and activated only by palmitate but not oleate. The latter difference might be due to the distinct cellular fate of these fatty acids. Whereas oleate is used to synthesize triglycerides which are stored without affecting cell viability [24], excess amounts of palmitate are only poorly incorporated into triglycerides and cause lipoapoptosis which in turn could lead to increased Ob-R shedding. Both, palmitate as well as staurosporine seem to directly influence Ob-R cleavage since a knockdown of ADAM10– or ADAM17-activity inhibited staurosporine and palmitate effects on sOb-R concentration. An also imaginable passive increase of Ob-R in the supernatant through an efflux across leaky membranes or formation of cell debris from dying cells should not be affected by the siRNA knockdown. Recently it was shown that mono-unsaturated fatty acids like oleate could prevent the lipotoxic effects of saturated fatty acids like palmitate [24]. Our results from oleate and palmitate co-incubation experiments not only affirm these findings but also let us speculate that the ratio of saturated to unsaturated fatty acids may influence the amount of shed Ob-R. Since only co-incubation with oleate but not the broad spectrum caspase inhibitor Z-VAD(OMe)-FMK reduced sOb-R to basal level and restored cell viability we suggest that similar to staurosporine caspase– or even apoptosis-independent mechanisms are of importance for effects seen after palmitate incubation. Lipotoxicity caused by elevated free fatty acids in tissues other than the adipose tissue [25], [26] and apoptosis for example of pancreatic beta cells occur during the manifestation of T1DM. Accordingly, our data suggest that strongly increased sOb-R levels observed in newly manifested T1DM [21], [22] could be induced by high levels of saturated FFAs like palmitate. Recently, it was demonstrated that high doses of leptin even without insulin administration normalize glucose and lipid metabolism in type I diabetic mice [53]–[55]. The high doses of leptin applied in these studies might have been necessary to overcome the inhibitory effect of high sOb-R levels in the type I diabetic mice. This hypothesis is supported by our findings that co-incubation of leptin with increasing amounts of sOb-R resulted in reduced leptin-mediated STAT3 phosphorylation in Ob-Rfl transfected HEK293 cells. Our data suggest that sOb-R levels which exceed a molar ratio of sOb-R to leptin of 2∶1 exhibit a predominant inhibitory effect on leptin signaling, a ratio that is frequently observed in catabolic diseases and early childhood [21], [22], [56]. As we could show that sOb-R mainly originates from short Ob-R isoforms, the signal transducing long Ob-R isoform in the mouse model described before might still occurred at the cell surface at sufficient amounts to mediate beneficial effects of the ligand. Therefore, decreasing Ob-R shedding by use of specific ADAM10 inhibitors perhaps could further improve the effects of a leptin mono therapy. Moreover, we demonstrated that co-expression of the short isoform Ob-R219.3 with Ob-Rfl impaired leptin signalling compared to cells transfected with Ob-Rfl only. These findings are in contrast to Bacart et al. [44], [57] who demonstrated that increasing amounts of the co-expressed short isoform Ob-Ra did not alter leptin signaling of the long receptor variant. However, our findings are supported by results of White et al. who demonstrated a modest repression on signaling of wildtype Ob-Rfl when they co-transfected increasing amounts of a truncated Ob-Rfl isoform [58]. In addition to modulation of shedding enzyme activity, regulation of Ob-R supply is a determining factor for the extent of sOb-R generation. Ligand-receptor interactions as well as receptor trafficking from ER to membrane could affect the amount of membrane-anchored Ob-R. Accordingly, we investigated to which extent high leptin levels and low grade inflammation- associated ER stress – two markers linked to obesity – take effect on leptin receptor availability for sOb-R generation. The exposure of Ob-R transfected cells to leptin concentrations in the physiological range led to decreased sOb-R levels. Bjorbaek et al. showed that leptin/leptin-receptor-complexes were internalized and transported to lysosomes for degradation [59]. This ligand-induced receptor internalization process probably accounts for the temporarily decrease of the plasma membrane Ob-R pool, resulting in decreased sOb-R levels as observed in the present study. The nucleoside antibiotic tunicamycin inhibits N-glycosylation of proteins [60] and increases the protein load of the ER finally leading to ER stress induction. We found a reduced leptin binding and an impaired up to blunted leptin signaling in Ob-Rfl transfected cells in parallel to reduced sOb-R levels after tunicamycin-induced ER stress. These findings support the hypothesis that increased ER stress may decelerate the turnover of Ob-R to the plasma membrane. Concomitantly, constitutive shedding could further decrease the amount of membrane Ob-R which might foster deterioration of leptin signaling. These hypotheses could complement recent findings describing a participation of the NF-κB (nuclear factor ‘kappa-light-chain-enhancer' of activated B-cells) as well as the JNK (c-Jun N-terminal kinase) signaling pathway in the development of ER stress- and obesity-associated leptin resistance [34]–[36]. An increase in PTP1B and SOCS3 protein levels that were reported previously to be inducers of leptin resistance [11], [30]–[35], [61] could accelerate the process of becoming insensitive to leptin on cellular level. We suggest that chronic ER stress in obese patients decreases the amount of sOb-R due to diminished membrane Ob-R expression. Accordingly, the determination of serum sOb-R concentrations may serve as a tool to monitor the development of a leptin resistant state. This suggestion was already supported by clinical data [16]. Future studies are necessary to clarify the exact mechanisms for reduced sOb-R concentrations in response to ER stress and high leptin levels. Such investigations should include the use of receptor endocytosis inhibitors like cytochalasin and the determination of ER stress inducing agent-linked effects on ADAM10 and ADAM17 activity. Since tunicamycin is a synthetic and not a physiological stimulus of ER stress, continuing investigations should be extended to animal models of obesity in order to uncover to which extent ER stress- associated effects on the leptin – leptin receptor axis are of importance *in-vivo*.

Our results base upon transient overexpression of Ob-R isoforms in HEK cells but not upon investigations in primary cells or non-transfected cell lines. This restriction is hardly to overcome as for example endogenous Ob-R expression of primary human hepatocytes or non-transfected HEK cells is to low for the analytical differentiation. Otherwise the contribution of individual isoforms to the overall amount of shed Ob-R and isoform-specific regulation can be only proven by a cell model with receptor transfection.

In summary, we provide novel insights into the regulatory mechanisms for sOb-R generation and its importance as a main player in the regulation and for the monitoring of leptin action: high sOb-R concentrations seem to directly modulate leptin effects thereby acting predominantly inhibitory. In contrast, low sOb-R levels, although having no direct influence on leptin action, could serve as one potential indicator for the development of a leptin resistant state in obesity.

## Supporting Information

Figure S1 **Normalisation of Ob-R shedding to Ob-R protein expression in whole cell lysates.** sOb-R levels in the supernatant of Ob-R transfected HEK cells were normalized to Ob-R-isoform specific protein expression in whole cell lysates.
(DOC)

Figure S2 **Metalloproteases are involved in Ob-R shedding.** sOb-R levels in the supernatant of Ob-R219.3 transfected HEK cells after incubation with GM6001 (25–100 µM) for 24 h and 48 h. sOb-R levels of GM6001-treated cells are displayed relative to those of non-treated cells. Data are presented as means ± SD of n≥3 experiments.
(DOC)

Figure S3 **Metalloproteases ADAM10 and ADAM17 are involved in PKC activated Ob-R shedding.** sOb-R levels in the supernatant of Ob-R219.3 transfected HEK cells that were pre-treated for 30 min with GI/GW (3 µM) and further incubated for 24 h without or with PMA (10 ng/ml). sOb-R levels of PMA-treated cells are displayed relative to those of non-treated cells. Data are presented as means ± SD of n≥3 experiments.
(DOC)

Figure S4 **Release of nucleosomes after induction of apoptosis with staurosporine.** (A) Western blot of Histon H3 in cytoplasmic fraction of HEK cells after staurosporine incubation. Ob-R transfected HEK cells were incubated with staurosporine (0.1–0.5 µM) for 24/48 h. Following this incubation Histon H3 was determined by western Blot analysis in the cytoplasmic fraction after subcellular fractionation. (B) Relative amount of nucleosomes in cell supernatants and cytoplasmic fraction after staurosporine incubation. Ob-R transfected HEK cells were incubated with 0.5 µM staurosporine for 24/48 h. Following this incubation nucleosome release was determined by Cell Death ELISA^PLUS^ (Roche) in cell supernatants and in the cytoplasmic fraction according to the instructions of the manufacturer. The amount of nucleosomes is displayed relative to nucleosome levels of non-treated cells. Data are presented as means ± SD of n = 3 experiments.
(DOC)

Figure S5 **Doxorubicin induced Ob-R shedding.** (A) Representative western blot of cleaved PARP after doxorubicin incubation. Ob-R transfected cells were incubated with doxorubicin (100–500 ng/ml) for 48 h. Following this incubation cleaved PARP in cell lysates was determined by western Blot analysis. (B) sOb-R levels in the supernatant of Ob-Rfl or Ob-R219.3 transfected cells after incubation with 500 ng/ml doxorubicin for 24/48 h. sOb-R levels of doxorubicin-treated cells are displayed relative to sOb-R levels of non-treated cells normalized to total protein content. Data are presented as means ± SD of n≥3 experiments.
(DOC)
