# Supplementary material for: Novel Regulatory Mechanisms for Generation of the Soluble Leptin Receptor: Implications for Leptin Action
Source: PLoS One. 2012 Apr 24;7(4):e34787. doi: 10.1371/journal.pone.0034787 (PMC3335825; doi:10.1371/journal.pone.0034787)
Supplement: Figure S4 — Release of nucleosomes after induction of apoptosis with staurosporine. (A) Western blot of Histon H3 in cytoplasmic fraction of HEK cells after staurosporine incubation. Ob-R transfected HEK cells were incubated with staurosporine (0.1–0.5 µM) for 24/48 h. Following this incubation Histon H3 was determined by western Blot analysis in the cytoplasmic fraction after subcellular fractionation. (B) Relative amount of nucleosomes in cell supernatants and cytoplasmic fraction after staurosporine incubation. Ob-R transfected HEK cells were incubated with 0.5 µM staurosporine for 24/48 h. Following this incubation nucleosome release was determined by Cell Death ELISAPLUS (Roche) in cell supernatants and in the cytoplasmic fraction according to the instructions of the manufacturer. The amount of nucleosomes is displayed relative to nucleosome levels of non-treated cells. Data are presented as means ± SD of n = 3 experiments. (DOC) [file pone.0034787.s004.doc]

**Figure S4**

**A**

**B**
