# Supplementary material for: Novel Regulatory Mechanisms for Generation of the Soluble Leptin Receptor: Implications for Leptin Action
Source: PLoS One. 2012 Apr 24;7(4):e34787. doi: 10.1371/journal.pone.0034787 (PMC3335825; doi:10.1371/journal.pone.0034787)
Supplement: Figure S5 — Doxorubicin induced Ob-R shedding. (A) Representative western blot of cleaved PARP after doxorubicin incubation. Ob-R transfected cells were incubated with doxorubicin (100–500 ng/ml) for 48 h. Following this incubation cleaved PARP in cell lysates was determined by western Blot analysis. (B) sOb-R levels in the supernatant of Ob-Rfl or Ob-R219.3 transfected cells after incubation with 500 ng/ml doxorubicin for 24/48 h. sOb-R levels of doxorubicin-treated cells are displayed relative to sOb-R levels of non-treated cells normalized to total protein content. Data are presented as means ± SD of n≥3 experiments. (DOC) [file pone.0034787.s005.doc]

**Figure S5**

**A**

**B**
